# Hearing impairment and daily-life fatigue: a qualitative study

**DOI:** 10.1080/14992027.2019.1597284

**Published:** 2019-04-28

**Authors:** Jack A. Holman, Avril Drummond, Sarah E. Hughes, Graham Naylor

**Affiliations:** a Hearing Sciences (Scottish Section), Division of Clinical Neuroscience, School of Medicine, University of Nottingham, Glasgow, UK;; b School of Health Sciences, University of Nottingham, Nottingham, UK;; c School of Medicine, Swansea University, Swansea, UK;; d South Wales Cochlear Implant Programme, Bridgend, UK

**Keywords:** Hearing loss, hearing impairment, fatigue, effort, daily-life

## Abstract

**Objective:** Hearing impairment is linked to increased fatigue, yet little is known about the real-world impact of this fatigue. This qualitative study investigated the experience of daily-life fatigue in people with a hearing impairment.

**Design:** Individual face-to-face semi-structured interviews were conducted. Thematic analysis was then used to analyse the data.

**Study sample:** Fourteen hearing impaired participants (aged 44–70 years) who varied in terms of hearing loss, hearing aid status, age and gender.

**Results:** The themes and sub-themes that emerged from the transcripts were: Fatigue (effort-driven fatigue, emotion-driven fatigue, breaks and recovery, the perceived relationship between hearing impairment and fatigue, and sleep), Effort (cognitive effort and physical effort), Coping Strategies (withdrawal, avoidance and planning), Relationships and Emotions, Hearing Aid Impact.

**Conclusions:** The study highlights that hearing impairment-related fatigue is experienced by many but not all, and to different extents. Hearing aids were weakly linked to a beneficial effect on fatigue. In addition to the more widely researched effort-driven fatigue, participants described fatigue linked to the negative emotions related to having a hearing impairment. These findings, in conjunction with the widespread utilisation of different coping strategies, demonstrate that the experience of fatigue is varied and likely dependent on personal factors and lifestyle.

## Introduction

Hearing loss affects ∼1 in 6 adults in the UK (Akeroyd, Foreman, and Holman, 2014). Fatigue is regularly described as comorbid within this large group of people (Bess and Hornsby 2014; Hetu et al. 1988), but there is little research in this area. The most often cited cause of increased fatigue in hearing impairment is that reduced audibility results in increased listening effort in order to maintain performance. Increased effort, in turn, is presumed to demand a greater drain on cognitive energy, manifesting as fatigue (McGarrigle et al. 2014). Evidence tends to suggest that people with hearing loss must exert more effort when listening than those without (Dwyer, Firszt, and Reeder 2014; Zekveld, Kramer, and Festen 2011). Whether this translates to an effect on fatigue, and if so, how, is still unclear (Hornsby, Naylor, and Bess 2016; McGarrigle et al. 2014).

Fatigue as a concept is complex and can be viewed in many different ways depending on the area of research or method of measurement. Fatigue may be assessed by self-report, behavioural, or physiological measures (McGarrigle et al. 2014). There is a large body of literature debating whether fatigue is best measured as a uni-dimensional or multi-dimensional construct (de Raaf, de Klerk, and van der Rijt 2013; Dittner, Wessely, and Brown 2004). In some studies, dimensions such as physical fatigue, mental fatigue, emotional fatigue and vitality/vigour are identified (Hockey 2013; Stein et al. 2004). However, other research using self-report outcome measures has found that people tend to respond to a single fatigue factor (Michielsen, De Vries, and Van Heck 2003). Additionally, fatigue may be either transient (momentary and task related) or long-term (i.e. not specifically task related). Transient fatigue is driven by demanding tasks whereas long-term fatigue is usually related to a health or environmental issue and arises when the experienced fatigue does not abate quickly after task demands cease. (Hockey 2013; Hornsby and Kipp 2016). In addition, research suggests that rather than fatigue being dependent entirely on depletion of cognitive energy, an individual’s motivation to invest cognitive resources towards a task moderates the amount of fatigue experienced in a given situation (Boksem, Meijman, and Lorist 2006; Chaudhuri and Behan 2000). Here fatigue is depicted as a signalling mechanism for directing resources away from unrewarding goals and towards beneficial goals (Hockey 2013). This study will investigate all of the aforementioned forms of fatigue from the perspective of individuals with hearing impairment.

Previous studies investigating fatigue and hearing loss have tended to use self-report outcome measures. The majority of this research has shown that people with an audiometric hearing loss report more fatigue than those without, however fatigue scores have tended to correlate with hearing handicap scores rather than audiometric thresholds (Alhanbali et al. 2017, 2018; Hornsby and Kipp 2016; Wang et al. 2018). Other studies have indirectly observed increased fatigue in people with hearing loss by reporting outcomes such as need for recovery after the working day (Nachtegaal et al. 2009). Such studies introduce the idea that the amount of communicative activity undertaken by an individual will affect the level of fatigue they experience. Additionally, the very small amount of research into the effect of wearing hearing aids on fatigue has shown an objective benefit (reduced performance decrement over time) but not a subjective benefit. Notably, objective and self-report measures of fatigue have been found not to correlate (Alhanbali et al. 2017; Hornsby 2013). Common to the research topic as a whole is the use of dissimilar outcome measures, some of which claim to measure fatigue directly, while others use indirect measures of fatigue.

The majority of research into this area has involved people with an audiometric hearing loss; however there are other hearing issues that are often co-morbid with hearing loss, particularly tinnitus, which may also affect fatigue (Asplund 2003). Therefore, in this study, hearing impairment as a whole was investigated. In order to investigate the everyday impact of hearing impairment on fatigue it is necessary to probe people’s experiences. For this reason a qualitative approach is appropriate (Pope and Mays 1995). Qualitative studies seek to provide an in-depth understanding of individuals’ experiences and behaviours and gain insight into the phenomenon of interest. Semi-structured interviews were chosen as the best way of obtaining insightful responses from participants that were not forced or leading, with scope for deviations into previously unobserved areas. Semi-structured interviews have been used to investigate the experience of fatigue in other disorders and diseases (Crosby et al. 2012; Yellen et al. 1997), and have shown that mental fatigue can be experienced by those with a hearing loss (Preminger and Laplante-Lévesque 2014).

Given the variability in methodology, lack of unanimity and the multi-faceted nature of fatigue, it is important to conduct research to clarify what the experience of fatigue is in everyday life for people with hearing impairments. As well as feelings of fatigue, this includes aspects of everyday life which may not always be expressly or solely stated by participants as being directly related to fatigue, but which could be indirectly related. The aim of this study, to investigate the impact of hearing impairment on fatigue in everyday life, was broken down into three main research questions:

1. Do all people with hearing impairment experience hearing impairment-related fatigue in everyday life?
2. How does hearing impairment-related fatigue manifest and what effects does it have on everyday life?
3. What effect does hearing aid fitting have on fatigue?

## Methods

### Design

All interviews were conducted face-to-face by author JH, who was a male Ph.D. student, in a closed room with no observers at the hospital department. Participants were informed that the study would form part of a Ph.D. thesis. Before the interview, an administrative questionnaire was completed as well as a pure tone audiometric hearing test. The questionnaire asked about hearing aid status, tinnitus and work status. After the interviews, participants were asked to list any other potentially fatiguing conditions they have. Interviews were recorded using a digital voice recorder. An interview question guide (Supplementary Appendix 1) was used to ensure coverage of all relevant research question areas. During the interviews, brief field notes were taken to outline the impressions of the interviews as extra information for stage 1 of the analysis process. The interviews were transcribed verbatim. The transcriptions were conducted by author JH for the first four interviews, and by a professional transcription service for the rest. The interviews ranged in duration from 18:36 to 42:51 min (average 28:12).

### Participant recruitment

Participants were hearing impaired adults (>18 years old) who had previously reported to NHS audiology with hearing problems, and were contacted via mail. Interviews took place over the course of three months. The participants were recruited using stratified purposeful sampling which involves sampling based on key variables within the initial major variable sample (Palinkas et al. 2015). Key variables considered during sampling were hearing aid status, degree of hearing loss, age and gender. These variables were known to the researcher prior to invitation and therefore could be used as part of the sampling process. Given that everyday activity could be important to the development and experience of fatigue in people with hearing impairment, a range of different lifestyles was sought based on work, social and physical activity. People in full-time work, part-time work and no work were included. Social activity and physical activity were also considered by attempting to recruit people of different ages, however these activity levels could not be obtained before the interview itself and were instead identified at the interview. The number of participants was decided by the point at which data saturation occurred (see Analysis section).

Fourteen participants took part in total (ten female). Their age range was 44–70 years (mean: 58). The worse-ear and better-ear four frequency average hearing loss ranges were 21–65 dBHL (mean: 42) and 16–61 dBHL (mean: 36), respectively. With regards to employment status, five were not in work, six worked part-time and three worked full-time (>30 h per week). Six of the participants wore bilateral hearing aids, four unilateral aids, and four had no hearing aids. No invited participants who replied subsequently declined to take part or dropped out.

This study was approved by the West of Scotland Research Ethics Service (18/WS/0030). All participants provided written informed consent (Table 1).

**Table 1.** Participant demographics.

	Study participants *N* = 14	
	Mean (standard deviation)	Range
Age	58 (7.1)	44–70
Better ear 4FAHL	36 (12.8)	16–61
Worse ear 4FAHL	42 (12.1)	21–65
	*N*	%
Gender		
Male	4	29
Female	10	71
Hearing aids		
No hearing aid	4	29
Unilateral	4	29
Bilateral	6	43
Work status		
Not in work	5	36
Part-time	6	43
Full-time	3	21

4FAHL: Four frequency average hearing loss (dBHL).

### Interview question guide

Questions were designed to answer the three research questions. In order to minimise any biasing of participants, the explicit questions regarding fatigue were embedded within wider questions about the impact of hearing impairment on everyday life which included prompts to further investigate responses. This made it possible to examine whether fatigue was brought up without prompting. At the outset it was stated to participants that the goal of the research was to study the impact of hearing impairment on everyday life. The responses to questions about the general impact of hearing impairment were also expected to be potentially insightful with regards to fatigue. In order to cover all important aspects of everyday life, regular prompts were used to ask about home, work and social life. At the end of the interviews, participants were debriefed regarding the exact goals of the research in addition to summarising the main points raised. This allowed the participants the opportunity to clarify or build on their responses. Participants were not followed up for further interviews or input regarding transcripts or findings.

The question guide went through several stages of development, beginning with one to one discussions between JH and GN. The guide was then presented in front of the wider research group in the same department to dissect and discuss. Finally, the guide was scrutinised by a researcher from out-with the department who had experience running semi-structured interviews in a different clinical population. The guide was then pilot tested once to ensure clarity and comprehensiveness. This resulted in a final question guide (Supplementary Appendix 1).

### Analysis

The anonymised, verbatim transcripts were uploaded for analysis using QSR International’s NVIVO 11 software (Melbourne, Australia). Thematic analysis was used to analyse the transcripts. Thematic analysis comprises of six stages designed to identify important themes that emerge from the data (Braun and Clarke 2006). These are (1) familiarisation with the data, (2) generation of initial codes, (3) searching for themes, (4) reviewing themes, (5) defining and naming themes, (6) producing the report. The stages are an iterative process which involves repeating previous steps in order to identify themes. The initial stages of analysis were undertaken concurrently with interviews in order to identify the point at which data saturation had occurred (i.e. no new information was forthcoming through changes to themes and subthemes). This decision was undertaken by researchers JH and GN. The majority of the generation of initial codes was completed by JH. Regular scrutinising of the transcripts occurred between JH and GN during the course of the study to discuss the ongoing development of themes, which involved moving backwards and forwards through all of the stages. At the stage of reviewing themes, the provisional themes were presented to the wider departmental research team along with extracts from the transcripts to gain further insight and potentially go back through the stages to make changes. Once there was consensus, the thematic analysis report of themes and sub-themes was created.

A key stage at the end of the process was the creation of the coding manual. The purpose of the manual is for other researchers to apply it to the transcripts in order to ascertain the validity and reliability of the results of the thematic analysis (Joffe and Yardley 2003). Based on the thematic analysis report, the coding manual explains, using definitions and examples, each of the themes. This manual (created by JH) was used by researcher SH to analyse a selection of the transcripts. SH is experienced in qualitative research but had not seen any of the data before this point. Any issues with the coding manual were noted and then later discussed with JH. A final version of the manual was then created, taking into account the suggested changes, and agreed upon by the researchers (Supplementary Appendix 2). This represents the ultimate results of the qualitative analysis.

## Results

**Figure 1.**
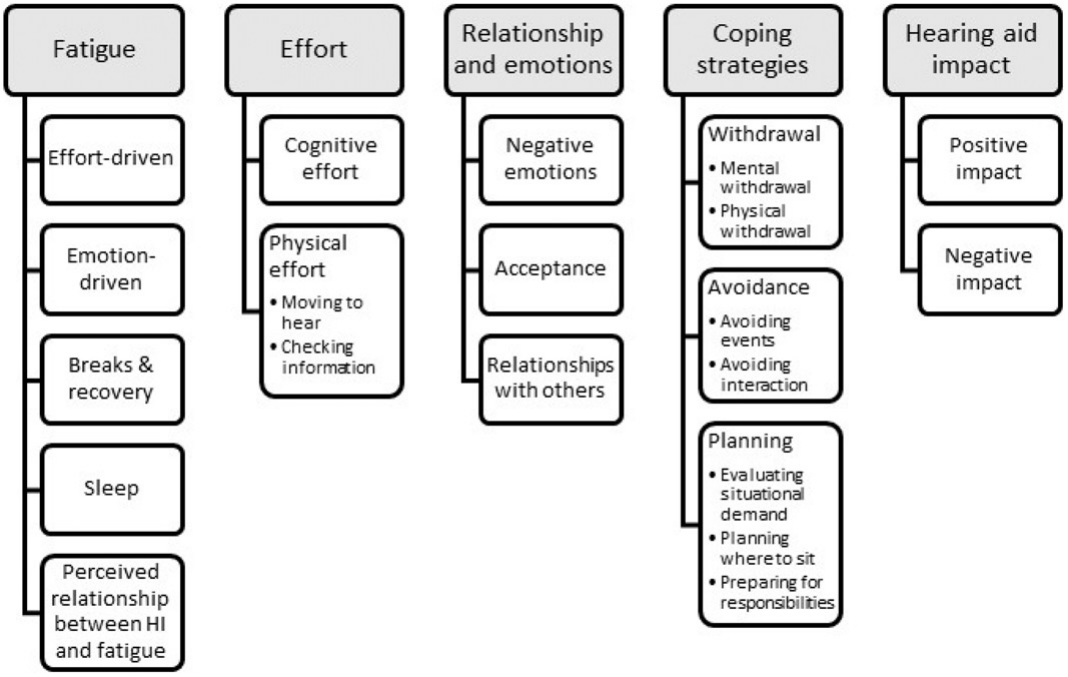
Hierarchy of themes arising from transcripts.

Five major themes were identified: fatigue, effort, relationships and emotions, coping strategies, and hearing aid impact (Figure 1). Together, they characterise the experience of hearing impairment-related fatigue in daily life, through direct reference to fatigue (theme 1) and indirect reference to fatigue through related experiences and behaviours (themes 2–5). In keeping with qualitative methodology, the relative frequency of occurrence of codes was not used or reported. The themes and subthemes are described in the following section, ordered by directness of relevance to the research questions and listed with the corresponding code from the coding manual. This study also used the Consolidated Criteria for Reporting Qualitative Research (COREQ) (Supplementary Appendix 3). COREQ is a 32 item checklist designed for explicit, comprehensive and transparent reporting of qualitative studies (Tong, Sainsbury, and Craig 2007).

### Theme 1: Fatigue

Participants spoke directly about the impact of hearing impairment upon fatigue in their everyday lives. Three main possible causes of fatigue were mentioned by participants: increased effort, negative emotions and (amongst participants experiencing tinnitus) sleep disruption. Additionally, participants described fatigue-specific need for breaks and recovery, and expressed their opinion on the perceived relationship between hearing impairment and fatigue. Descriptions of fatigue were most common in work and social situations. Through the interviews, it became apparent that different participants undertook different levels and types of activity during an average week, both in terms of occupation (Table 2) and social activity.

**Table 2.** Participant characteristics.

	Age (years)	Gender	HA status	Work status	Tinnitus	4FAHL BE	4FAHL WE
P001	58	Female	Unilateral	Part-time	No	47.5	55
P002	44	Male	None	Not in work	Yes	26.25	27.5
P003	51	Female	Unilateral	Part-time	Yes	25	27.5
P004	57	Female	Unilateral	Part-time	Yes	25	53.75
P005	60	Female	Bilateral	Full-time	Yes	28.75	52.5
P006	70	Male	Unilateral	Not in work	No	42.5	46.25
P007	47	Female	Bilateral	Part-time	Yes	42.5	47.5
P008	53	Male	Bilateral	Full-time	Yes	46.25	47.5
P009	64	Female	None	Not in work	No	16.25*	21.25*
P010	56	Male	Bilateral	Part-time	No	37.5	37.5
P011	61	Female	None	Not in work	No	61.25	65
P012	65	Female	Bilateral	Part-time	No	35	35
P013	57	Female	None	Full-time	No	41.25	42.5
P014	62	Female	Bilateral	Not in work	No	25	28.75

4FAHL: Four frequency average hearing loss better ear (BE) worse ear (WE) (dBHL).

*Participant had high-frequency averages (4 and 8 kHz) of 55 and 62.5.

#### F1: Effort-related fatigue

Participants often described feelings of tiredness or weariness that arose as a direct result of the need for extra concentration and mental effort when listening. This was most usually described as a general or mental tiredness. While certain situations were labelled as tiring, the fatigue was often stated as being felt upon resolution of the event or at the end of the day, with direct causation being attributed by participants to the difficulties caused by their hearing impairment.

I do find it tiring [hearing impairment] because I feel that I've got to put additionally an extra focus on when people are speaking, maybe it's because I'm in like a fairly responsible job you know, it's not as if I just can’t not be seen to not listen you know I've got to really listen and understand people and carve out my own instructions for my own workload. So, by the end of a working day for me or any day in general I feel quite tired with it because I feel I'm having to focus more, lip read more or you know face people more that sort of thing and I would say it adds a certain amount of stress to you (P008).

It was also suggested that increased physical effort due to a hearing impairment was sometimes responsible for fatigue. It is therefore meaningful to draw a distinction between concentration/focus and movement or repetition. Positioning and movement in order to hear, as well as the need for repetition, were the main reasons for effort-related fatigue that was not exclusively linked to cognitive effort.

Having to concentrate more and going in and out of the room when I’m doing something else is tiring (P004).

I’m trying to do a new job just now and trying to get - the guy that’s showing me stuff whispers, so I have to say a couple of times, what was that or say that again. That’s extra effort, so I’m feeling a bit exhausted now with just learning stuff (P001).

#### F2: Emotion-driven fatigue

In addition to fatigue resulting from increased effort, participants mentioned that sometimes fatigue was the result of negative emotions associated with participants’ experiences of living with a hearing impairment. Emotion-driven fatigue was reported predominantly as a consequence of frustration and stress experienced during conversation with others. These fatiguing emotions were suggested to be a direct result of not being able to hear, or to occur indirectly due to social pressure (i.e. the requirement to take an active part in social events). There was also an example of fatigue directly linked to tinnitus, due to the negative emotions resulting from the perceived noise.

Frustration I think does make me tired, sometimes I find myself losing interest in the conversation, I find myself losing interest in the company I'm with (P010).

It [social situations] can be a wee bit, mentally tiring. Like I say, if I’m in a group of people I don’t really know, it can – I get a wee bit stressed (P002).

Yes, double check, so you know it is frustrating as a person when you have that and it is tiring because it's always in the back of your mind psychologically you know, you're thinking people think I'm not interested or they're not showing any interest so you know, you probably think too much and become paranoid in a way (P008).

#### F3: Breaks and recovery

Some participants gave accounts of challenging listening situations which resulted in them taking breaks or having spells of recovery. These accounts, while not mentioning fatigue directly, suggest that hearing impairment is a significant contributory factor to the fatigue experienced by participants.

And it’s almost like after that you want to just go into a total quiet place and just go right okay, give me five minutes (P003).

#### F4: Sleep

Difficulty going to sleep was described as a cause of fatigue the next morning by those participants who stated that they experience tinnitus. For some, this tiredness persisted throughout the next day, but others mitigated it.

At night I’m not sleeping with it [tinnitus] as it seems to be worse at night (P004).

It would make me certainly start off the day tired, but because you work with children you’ve got to – you’re always very, very aware of not bringing your home into your school… So the professionalism kicks in at that stage and you just have to get on with it (P003).

#### F5: The perceived relationship between hearing impairment and fatigue

Throughout the interviews, the experience of hearing impairment-related fatigue across participants varied considerably. Some participants explained that hearing impairment-related fatigue was a major issue that they faced. A few participants did not experience it at all. Others felt that it contributed to their tiredness but that it was either not the major cause of fatigue in their lives or that they had never thought about the possibility of their hearing impairment contributing to fatigue. Therefore it seems likely that hearing-related fatigue is not experienced by all people with a hearing impairment.

I'm sure there's a cross link maybe between the two together you know, it's maybe not just all down to hearing all the time it's probably a bit of demands of the job and you're trying to deal with that as well (P008).

I: Were there any levels of tiredness in those situations? P: I don’t know, I’ve never put it together. It’s a bit draining (P007).

Probably the most tiring thing for me in my everyday life is a group conversation (P008).

### Theme 2: Effort

Increased effort was mentioned directly as one of the most common reasons for hearing impairment-related fatigue. Participants, however, went into more detail about how hearing impairment results in an increased effort, which may, therefore, be relevant to its impact on fatigue.

#### E1: Cognitive effort

##### E1.1: Concentration

Participants regularly discussed that extra mental effort was needed in conversation with others due to their hearing impairment. Added concentration and focus on listening was stated as well as the need to watch lips, facial expressions and body language in order to maintain conversations.

Yes, but I’ve still got to concentrate and look at people more. Now, when people speak to me, I look at their face. Before, you could have talked to me and if I was writing something then I wouldn’t bother looking at them. Now, I have to look at people when they talk to me (P005).

#### E2: Physical effort

##### E2.1: Moving to hear

Participants stated that in order to aid comprehension they would move to hear clearer. This was sometimes by walking closer to the person speaking, changing room or leaning closer.

And then as they were trying to speak to me, if they were over there, I would say hold on a wee minute. And I would be right up close to them so I could pick up what they were saying (P012).

##### E2.2: Checking of information

Participants reported regularly needing to check the information they were receiving. Checking of information was most commonly a verbal request for repetition. Re-listening to electronic messages such as voicemail was also mentioned.

…having to maybe double check any messages that are on the phone, rather than having to just pick up on the first case scenario you know I'm always judging myself did he say that or did she say that (P008).

### Theme 3: Relationships and emotions

The impact of hearing impairment on relationships and emotions was probed specifically during the interviews. Participants regularly reported that hearing impairments have had a negative impact on their personal relationships and emotions. This is distinct from the emotion-driven fatigue identified in theme 1.

#### R1: Negative emotions

Emotions and feelings experienced as a result of hearing impairment were overwhelmingly negative. Emotions that were expressed ranged from frustration and embarrassment to sadness and resentment and were suggested to result from the limitations imposed on everyday life by the hearing impairment.

It does make me sad that this is it and it’s not going to get any better (P005).

I’m missing bits you know and that’s very frustrating (P009).

#### R2: Acceptance

While there were no positive emotions that arose from having a hearing impairment, there were some statements that demonstrated a level of acceptance of the problem.

I’m getting used to it, so it doesn’t really bother me now (P001).

The closest that any participant got to a positive statement was that hearing very little meant that they got a better night’s sleep than their partner.

#### R3: Relationships with others

Hearing impairment and the consequent reduction in conversational ability can result in emotions such as frustration and anger for both the person with hearing impairment and others in the conversation. Participants cited a lack of empathy and understanding from friends, family and colleagues as one of the main causes of friction in relationships. This is the extension of the negative emotions that hearing impairments can cause, into psychosocial consequences.

My son gets annoyed with me because when he phones me up and I give him all the wrong answers (P011).

There can be quite a point of blowing up at each other but I can understand where she’s coming from but I don’t think she understands how I feel about it (P008).

### Theme 4: Coping strategies

Participants revealed several different coping strategies linked to difficult listening situations. The strategies were sometimes described specifically as addressing fatigue and effort, however, strategies were on occasion reported with no specific insight into the reason for their utilisation. Participants reported mentally or physically avoiding or withdrawing from difficult listening or communication situations, and a need to plan and prepare for listening tasks. Coping strategies were most commonly reported for work and social situations.

#### C1: Withdrawal

##### C1.1: Mental or emotional withdrawal

A strategy of mental withdrawal was often mentioned as being utilised in order to deal with experienced fatigue and effort. In conversational situations, particularly group conversations, participants would stop engaging in listening. This was usually described as a response to the situation being “not worth it” anymore. Sometimes this would be accompanied by nodding along so as to appear engaged in the conversation.

The noise level goes up and up and up and if I’m talking to you, I can’t hear you clearly and I’d miss out on every third word you say. Then I get frustrated and start switching off (P006).

I can find myself getting tired with getting back to what I said earlier on, I have to step back a bit from the conversation sometimes (P010).

##### C1.2: Physical withdrawal

Occasionally participants described physically removing themselves from a challenging situation, rather than mental withdrawal. Again this tended to be due to a decision that the conversation was not worth the bother anymore, particularly in noisy group environments.

I find it difficult and I’ll think, ‘Why am I doing this?’ It’s different when you’re in a car and you can’t just open the door and get out. I make the excuse of going for a cup of tea or start mingling (P006).

#### C2: Avoidance

##### C2.1: Avoiding events or situations

As well as withdrawing, participants also pre-emptively avoided events due to expected problems resulting from their hearing impairments. The reasoning for avoidance was the same as for withdrawing, but avoidance was a strategy that was introduced ahead of time. Some found this to be a dilemma between their dislike for challenging situations and their wish to maintain a social life or not offend people.

I think it could be a combination thing, you know if I knew where we were going was going to be noisy, things like ceramic floors and just a general din and I was really tired I might decline the offer, I might decline the offer, it is unlikely because I'm quite a social person (P009).

I feel stupid, you know if somebody is saying something and I don't know how to answer it so I try to avoid situations where I'm going to be in close contact with people (P013).

##### C2.2: Avoiding interaction with others

A more specific occurrence that was raised by some participants was the decision not to engage with others when they were already in a social situation. This was usually at group events such as restaurant meals or weddings where there are multiple potential conversational options with a decision already having been made to attend. It would then be decided that to avoid negative effects such as increased fatigue or embarrassment, certain people would not be engaged with.

But, but if you’re in a group, you can choose, so if I was at a wedding I would pick out people that I was going to have a conversation with (P003).

#### C3: Planning

##### C3.1: Evaluating situational demand

Participants detailed different ways in which they plan ahead of time to mitigate challenging listening situations. Examples include identifying what location to go to, who was going to be there, and whether or not to wear hearing aids. Planning, in general, could plausibly be interpreted as additional effort rather than as a coping strategy, however, planning was almost always described as a way to avoid negative outcomes rather than being a negative outcome in itself.

I analyse who’s going to be there (P001).

When I go out with my husband and this friend he has a hearing loss so we’re inclined to go for places that we know are not going to be noisy (P009).

##### C3.2: Planning where to sit

A specific but common example of planning deemed necessary due to a hearing impairment was deciding where to sit. Participants would position themselves so that they could see faces and improve audibility.

There’s a lot of thought that goes into it before; before I meet the person and we get to the venue, I scan the table for the best place to sit (P004).

##### C3.3: Preparing for work/responsibilities

Occasionally it was mentioned that having a hearing impairment resulted in participants putting in additional efforts ahead of time in order to make the situation easier, usually a work scenario or event for which they were responsible.

I’d try to prepare in advance and build on names, who they were etc. I would do a lot of preparation that other people wouldn’t do in order to help myself (P004).

### Theme 5: Hearing aid impact

While not all participants had hearing aids, those that did described the impact hearing aids have on their everyday life and how they affect the outcomes from having a hearing impairment.

#### A1: Positive impact of hearing aids

Hearing aids were primarily described as improving audibility. This, in turn, led to a general feeling of improved well-being as well as improved social participation and reduced effort and fatigue. Although never stated unprompted, when asked specifically, every participant who experienced hearing impairment-related fatigue said that wearing hearing aids lessened their experienced fatigue. Improved conversational ability was one of the most common unprompted responses. Participants struggled to report on the relative impact of hearing aids on fatigue that arises due to effort, emotion or sleep disruption.

If I’ve not got them, I won’t put myself in the situation. I’ve always got at least one in. I wouldn’t dream of going out without them, I would hate it (P007).

Well it makes it a bit clearer because I can actually hear music and people talking the same as, I could only hear the music and not hear people but now I can hear both (P012).

#### A2: Negative impact of hearing aids

Participants also mentioned that hearing aids have negative effects on their everyday lives. The negative effects consisted of distorted and unpleasant sounds, physical pain when wearing them, amplification of background noise and embarrassment to be seen wearing them. There were no specific negative effects mentioned in relation to fatigue.

No, they actually made it [conversation] worse. The clanking of the cutlery or glasses on the table when I was out for a meal was booming. Background music as well, if it’s to one side of me then it’s particularly bad (P004).

## Discussion

Hearing impairment has been shown in previous literature to be linked to increased levels of fatigue. Directly through reference to fatigue, and indirectly through reference to concomitant emotions and behaviours, this study addresses the three research questions: (1) Do all people with hearing impairment experience hearing impairment-related fatigue in everyday life? (2) How does hearing impairment-related fatigue manifest and what effects does it have on everyday life? (3) What effect does hearing aid fitting have on fatigue? This is achieved with the findings that:

1. Many, but not all, participants were aware of experiencing hearing-impairment related fatigue.
2. Hearing impairment-related fatigue can result from increased effort, negative emotions or disruption to sleep. There is great variation among individuals regarding the impact their hearing impairment has on their fatigue, however, altered behaviour and utilisation of coping strategies are some of the described effects.
3. Hearing aid fitting does seem to have a beneficial impact on fatigue, however, the magnitude of benefit is not clear.

The finding that hearing-impairment related fatigue is not experienced by all people matches the reporting of fatigue by some but not all participants in previous research into hearing impaired populations (Hetu et al. 1988). Most current research into hearing impairment and fatigue focus on an increased effort in the form of concentration as the main origin of hearing-impairment related fatigue (Hornsby 2013; Hornsby et al. 2016; Preminger and Laplante-Lévesque 2014). This finding is echoed in this study, and is consistent with current conceptualisations of listening effort described by the Framework for Understanding Effortful Listening (FUEL) (Pichora-Fuller et al. 2016) and Kahneman’s Capacity Model of Attention (Kahneman 1973). Meanwhile, the sub-themes of “moving to hear” and “checking of information” within the theme of “effort” show that exerted effort can be in forms other than just cognitive effort, and that its effects as a precursor of fatigue can continue beyond the end of a conversation.

The finding that people become emotionally fatigued and wish they were not in a given situation is consistent with the psychosocial consequences of living with hearing loss (Chia et al. 2007; Ciorba et al. 2012). Importantly, negative emotion as a cause of fatigue has been reported in the wider literature, particularly in working populations through interpersonal conflict (Barnes and Van Dyne 2009; Cropanzano, Rupp, and Byrne 2003). Until now emotion-driven fatigue has received little attention in hearing impairment research.

The reporting of tinnitus being linked to increased fatigue through difficulty sleeping and negative emotions caused by the sound during the day is supported by previous findings. These identified that tinnitus causes “daytime sleepiness” through disturbance to sleep, in addition to the tiring effect of the tinnitus sound itself. (Asplund 2003; Cronlein et al. 2007). With regards to how sleepiness is represented in the wider fatigue literature, while current research suggests that the systems underpinning physical or mental fatigue and sleepiness may be fundamentally different in terms of origin, they are each subject to the same central regulation through the compensatory strategy of performance protection in task situations (Drummond et al. 2004; Drummond, Gillin, and Brown 2001).

One important effect of increased fatigue on daily life shown in this study is the need for participants to deploy coping strategies. Qualitative research has previously highlighted some of the same coping strategies and psychosocial consequences (Heffernan et al. 2016; Hughes et al. 2018), although with reference to outcomes such as listening effort rather than fatigue. Additionally, motivation has been shown to have a role in the choice of coping strategy (Picou, Aspell, and Ricketts 2014). The varied utilisation of coping strategies could lead to diverse experiences of fatigue, just as do varying levels of physical, social and work activity (Oerlemans and Bakker 2014; Park et al. 2001; Ravesloot et al. 2016). The conscious use of certain coping strategies by some participants to alleviate or avoid fatigue, while other participants are unsure exactly why they demonstrate the same strategies, could highlight a subconscious avoidance of fatigue.

The fact that all participants who experienced hearing impairment-related fatigue and used hearing aids stated that the hearing aids helped, suggests that there is some benefit with respect to reduction of fatigue. As participant accounts of fatigue reduction with hearing aid use were only forthcoming after further probing from the researcher, this suggests that the benefit to fatigue could be either small in magnitude or less significant in the face of the other capability benefits hearing aids provide. This positive but potentially small role of hearing aids in relieving fatigue matches the current literature (Alhanbali et al. 2017; Hornsby 2013).

### Limitations

Purposeful sampling was intended to include participants who differed widely with respect to age, hearing aids status, occupational status and levels of hearing loss. In this respect, the obtained age range of 26 years was somewhat limited. The inclusion of younger participants might have introduced different lifestyles to the study group, whilst older participants may have experienced increased fatigue due to increasing likelihood of health issues and changes in cognition. Likewise, participants with a more profound hearing loss might also have exhibited a more pronounced effect of fatigue. Participants were recruited from a database of participants who had previously taken part in at least one study at the department. This might have led to a bias towards participants who are either very outgoing, have a higher motivation or who have lifestyles that allow them to take time out to help in research, i.e. not the highest level of demand or fatigue. During analysis, one researcher generated the majority of the initial codes, after which point the second researcher began assessing the texts with those codes in mind to change or adapt. This is not the method Braun and Clarke (2006) suggested, where both researchers would develop initial codes separately before comparing. However, we did include an additional stage at the end of analysis involving deductive analysis to assess reliability and validity (Joffe 2012), but the deviation from Braun and Clarke must be noted.

## Conclusions

This is the first study of its kind to offer direct insight into the daily-life fatigue experienced by people with a hearing impairment. The findings indicate that fatigue is an issue experienced by many, but not all, people with a hearing impairment. There is a strong emotion-driven aspect to fatigue in addition to the more commonly discussed effort-driven fatigue. There is widespread utilisation of coping strategies by individuals to mitigate the impact of hearing impairment. Some strategies are utilised purposely to moderate fatigue and effort, whilst some strategies are undertaken automatically with limited insight into how they impact on fatigue and listening effort. The different levels of impact that hearing impairment has on fatigue may be partially explained by the diversity of lifestyles (i.e. different levels of work and social activity) and differing utilisation of coping strategies. Future research is needed to investigate the fatigue associated with specific listening activities, and how hearing-related fatigue is impacted by an individual’s motivation to engage in given situations. Additionally, given that the extent of fatigue attributable to hearing impairment varies widely amongst people, more research is needed to investigate the impact hearing aid fitting has on fatigue.
